# Widening Educational Disparities in Premature Death Rates in Twenty Six States in the United States, 1993–2007

**DOI:** 10.1371/journal.pone.0041560

**Published:** 2012-07-20

**Authors:** Jiemin Ma, Jiaquan Xu, Robert N. Anderson, Ahmedin Jemal

**Affiliations:** 1 Surveillance Research Program, American Cancer Society, Atlanta, Georgia, United States of America; 2 Mortality Statistics Branch, National Center for Health Statistics, Centers for Disease Control and Prevention, Hyattsville, Maryland, United States of America; Chancellor College, University of Malawi, Malawi

## Abstract

**Background:**

Eliminating socioeconomic disparities in health is an overarching goal of the U.S. Healthy People decennial initiatives. We present recent trends in mortality by education among working-aged populations.

**Methods and Findings:**

Age-standardized death rates and their average annual percent change for all-cause and five major causes (cancer, heart disease, stroke, diabetes, and accidents) were calculated from 1993 through 2007 for individuals aged 25–64 years by educational attainment as a marker of socioeconomic status, using national vital registration data for 26 states with consistent educational information on the death certificates. Rate ratios and rate differences were used to assess disparities (≤12 versus ≥16 years of education) for 1993 through 2007. From 1993 through 2007, relative educational disparities in all-cause mortality continued to increase among working-aged men and women in the U.S., due to larger decreases of mortality rates among the most educated coupled with smaller decreases or even worsening trends in the less educated. For example, the rate ratios of all-cause mortality increased from 2.5 (95% confidence interval (CI), 2.4–2.6) in 1993 to 3.6 (95% CI, 3.5–3.7) in 2007 in men and from 1.9 (95% CI, 1.8–2.0) to 3.0 (95% CI, 2.9–3.1) in women. Generally, the rate differences (per 100,000 persons) of all-cause mortality increased from 415.5 (95% CI, 399.1–431.9) in 1993 to 472.7 (95% CI, 460.2–485.2) in 2007 in men and from 165.4 (95% CI, 154.5–176.2) to 256.2 (95% CI, 248.3–264.2) in women. Disparity patterns varied largely across the five specific causes considered in this study, with the largest increases of relative disparities for accidents, especially in women.

**Conclusions:**

Relative educational differentials in mortality continued to widen among men and women despite emphasis on reducing disparities in the U.S. Healthy People decennial initiatives.

## Introduction

Eliminating health disparities among different segments of the U.S. population defined in terms of socioeconomic status (income, education, insurance status, etc.), race/ethnicity, residence, sex, and sexual orientation has been an overarching goal of the decennial Department of Health and Human Services' Healthy People Initiative, which began in 1979 [1], [2]. Previous studies that have examined temporal trends in mortality disparities by socioeconomic status found widening rather than narrowing of the disparities [3]–[10]. In our previous report, we investigated individual-level socioeconomic disparities in mortality using national vital statistics and noted increasing educational disparities from 1993 through 2001 for all-cause and several major causes of death in both men and women [11].

In this study, we updated the data for 26 states included in our previous analysis by six additional data years (1993–2007) to present contemporary patterns of educational disparities in mortality rates from all causes and five major causes (cancer, heart disease, stroke, diabetes, and accidents). The 26 states were chosen because they are the only states that constantly used one version of the death certificate (1989-version) throughout the study period. The analyses were restricted to age 25–64 years because educational attainment information is more complete and provides a better index of socioeconomic position in this age group than at older ages [12], [13]. Notably, deaths occurring in this age interval are of societal importance as they tend to affect individuals and families with dependent children and/or other family members [14]. Education is used as marker of socioeconomic status.

## Methods

Mortality data from 1993 through 2007 were obtained from the National Vital Statistics System (NVSS) administered by the Centers for Disease Control and Prevention's National Center for Health Statistics (NCHS). Underlying causes of death were classified according to the selection and coding rules of the Ninth Revision of the International Classification of Diseases (ICD-9) [15] for deaths recorded between 1993 and 1998 and according to the Tenth Revision of the International Classification of Diseases (ICD-10) [16] for deaths recorded between 1999 and 2007. The ICD codes for the five major causes of death among men and women aged 25–64 years are as follows: cancer (ICD-9: 140–208; ICD-10: C00-C97); heart disease (ICD-9: 390–398, 402, 404, 410–429; ICD-10: I00-I09, I11, I13, I20-I51); stroke (ICD-9: 430–434, 436–438; ICD-10: I60-I69); diabetes (ICD-9: 250; ICD-10: E10-E14); and accidents (ICD-9: E800-E869, E880-E929; ICD-10: V01-X59, Y85-Y86). Less than 1% of deaths were recorded with unknown cause of death.

Educational attainment information for the deceased, which was recorded as number of years of schooling, has been included on the standard U.S. death certificate since 1989. However, most states did not collect this information systematically until 1993. In 2003, the question about educational attainment on the standard death certificate changed from number of years of elementary/secondary or college education to the highest degree received [17]. Although overall there is consistency between the levels in the old and new education items, some specific categories, such as 13–15 years of schooling on the 1989 certificate and some college credit on the 2003 certificate do not match up very well [18]. In addition, the gradual adoption of the new certificate (4 states in 2003 and 23 states and Washington DC in 2007) makes trend analysis difficult. Therefore, we restricted the analyses of mortality trends to deaths occurring in the 26 states (Alabama, Alaska, Arizona, Arkansas, Colorado, Hawaii, Illinois, Indiana, Iowa, Kentucky, Louisiana, Maine, Maryland, Massachusetts, Minnesota, Mississippi, Missouri, Nevada, North Carolina, North Dakota, Pennsylvania, Tennessee, Vermont, Virginia, West Virginia, and Wisconsin) that had not yet implemented the new standard certificate as of 2007. In these 26 states, a total of 3,580,612 deaths to persons aged 25–64 occurred between 1993 and 2007, which comprised 43.6% of the total deaths in the entire U.S recorded in this age interval during this period. About 4% of decedents with missing education information during 1993–2007 were excluded from the analysis.

Population estimates were obtained in a custom-designed tabulation from the U.S. Bureau of Census (Victor Valdisera, Housing and Household Economic Statistics Division, U.S. Bureau of Census, personal communication, 2010). These data were based on the Annual Social and Economic Supplement to the Current Population Survey (CPS), in which people's educational attainment was recorded as their highest degree obtained.

Educational attainment was classified into three categories: ≤12 years of schooling, 13–15 years of schooling, and ≥16 years of schooling. Age-standardized death rates from all causes combined and from the five major causes were calculated by race/ethnicity (all races combined, non-Hispanic white, and non-Hispanic black), sex, educational attainment, and calendar year, using the 2000 U.S. population standard within the age range 25–64 years. Temporal trends in death rates were assessed by fitting log-linear joinpoint regression models that allowed at most two change points, using Joinpoint Regression Program, Version 3.4.2 software [19]. The resulting trends of various time periods were described by the slope of the fitted line segment or the annual percent change (APC). We also presented the overall trends from 1993 through 2007 using the Average Annual Percent Change (AAPC), which is the weighted average of the APCs, with the weights equal to the length of each line segment. Changes in relative (rate ratio [RR]) and absolute (rate difference (RD]) educational disparities from 1993 to 2007 were assessed by comparing the age-standardized death rates in the least educated group (≤12 years of education) to those in the most educated group (≥16 years of education). The 95% confidence intervals (CI) for these disparities were calculated based on the formulas provided by Greenland and Rothman [20]. To understand the extent to which temporal changes of educational distribution in the population may have affected disparity trends, sensitivity analyses were performed using relative index of inequality (RII) and slope index of inequality (SII) as disparity measures.

## Results

**Figure 1:**
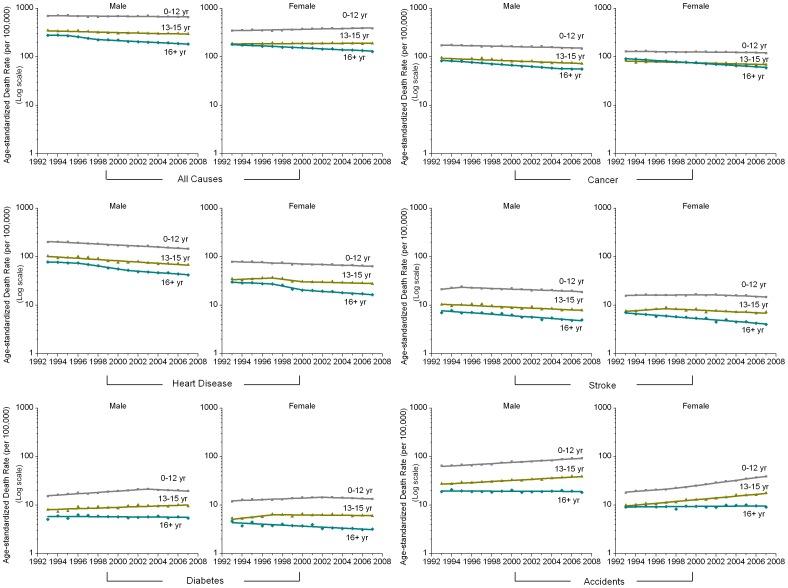
Trends in Age-Standardized Death Rates from All Causes and Five Major Causes by Educational Attainment in 26 U.S. States, 1993–2007.

Table 1 and Figure 1 show trends in death rates from all causes combined from 1993 through 2007 for 26 states among men and women aged 25–64 years (all races combined), in relation to educational attainment. Among men, death rates from all causes decreased for each educational attainment category, with the annual percentage decrease ranging from 0.4% for men with ≤12 years of education to 3.0% for men with ≥16 years of education. In contrast to what was observed for men, death rates increased by 0.9% for women with ≤12 years of education, decreased by 2.2% per year for women with ≥16 years of education, and remained unchanged for women with 13–15 years of education.

**Table 1:** Trends in Age-Standardized Death Rates from All Causes and Five Major Causes by Education among Men in 26 U.S. States, 1993–2007.

	Male							Female						
	Trend 1		Trend2		Trend 3		AAPC	Trend 1		Trend2		Trend 3		AAPC
	Years	APC	Years	APC	Years	APC		Years	APC	Years	APC	Years	APC	
All Causes														
Education: All	1993–1995	−0.9	1995–1998	−3.7*	1998–2007	−1.0*	−1.5*	1993–2007	−0.7*					−0.7*
≤12 years	1993–2007	−0.4*					−0.4*	1993–2007	0.9*					0.9*
13–15 years	1993–2007	−1.1*					−1.1*	1993–2007	0.2					0.2
≥16 years	1993–1995	−1.3	1995–1998	−6.3*	1998–2007	−2.3*	−3.0*	1993–2007	−2.2*					−2.2*
Cancer														
Education: All	1993–2007	−2.3*					−2.3*	1993–2007	−1.8*					−1.8*
≤12 years	1993–2007	−1.0*					−1.0*	1993–2007	−0.5*					−0.5*
13–15 years	1993–2007	−1.8*					−1.8*	1993–2007	−1.2*					−1.2*
≥16 years	1993–2005	−3.3*	2005–2007	−1.1			−3.0*	1993–2007	−2.9*					−2.9*
Heart Disease														
Education: All	1993–1997	−2.7*	1997–2000	−6.2*	2000–2007	−2.4*	−3.3*	1993–2007	−3.4*					−3.4*
≤12 years	1993–2007	−2.4*					−2.4*	1993–2007	−1.6*					−1.6*
13–15 years	1993–2007	−2.9*					−2.9*	1993–1997	2.2	1997–2000	−5.8	2000–2007	−1.2*	−1.2
≥16 years	1993–1996	−1.8	1996–2001	−6.8*	2001–2007	−3.2*	−4.2*	1993–1997	−2.0*	1997–2000	−9.3*	2000–2007	−3.0*	−4.1*
Stroke														
Education: All	1993–1995	2.4	1995–2002	−3.2*	2002–2007	−1.8*	−1.9*	1993–2007	−2.0*					−2.0*
≤12 years	1993–1995	4.5	1995–2007	−1.7*			−0.8	1993–2002	0.3	2002–2007	−1.9*			−0.5
13–15 years	1993–2007	−2.1*					−2.1*	1993–1997	3.1	1997–2007	−2.1*			−0.7
≥16 years	1993–2007	−3.3*					−3.3*	1993–2007	−3.6*					−3.6*
Diabetes														
Education: All	1993–2003	1.5*	2003–2007	−1.8			0.6	1993–2001	0.1	2001–2007	−2.6*			−1.1*
≤12 years	1993–2003	3.2*	2003–2007	−2.3			1.6*	1993–2002	1.9*	2002–2007	−1.6			0.7*
13–15 years	1993–2007	1.6*					1.6*	1993–1997	5.6*	1997–2007	−0.4			1.3
≥16 years	1993–2007	−0.3					−0.3	1993–2007	−2.3*					−2.3*
Accidents														
Education: All	1993–2002	0.9*	2002–2007	3.7*			1.9*	1993–1998	1.7*	1998–2007	4.7*			3.6*
≤12 years	1993–2007	2.8*					2.8*	1993–1998	3.8*	1998–2007	6.5*			5.5*
13–15 years	1993–2007	2.6*					2.6*	1993–2007	4.3*					4.3*
≥16 years	1993–2007	−0.2					−0.2	1993–2007	0.4					0.4

Abbreviations: APC, Annual Percent Change; AAPC, Average Annual Percent Change.

* P<0.05.

Table 1 and Figure 1 also present trends in death rates from 1993 through 2007 by educational level for five major causes of death in men and women (all races combined). For all the three major causes (cancer, heart disease, and stroke) for which the death rates were decreasing in the U.S. general population, the most educated group (≥16 years) experienced the largest decrease in death rates during the 15-year interval in both genders, while the other two less educated groups (≤12 and 13–15 years) experienced a much smaller or a non-significant decrease. For example, the death rate from cancer decreased by 3.0% per year from 1993 through 2007 among men with ≥16 years of education, compared to 1.8% and 1.0% per year for men with 13–15 years and ≤12 years of education, respectively. For the death rate from stroke in women, a significant decrease (3.6% per year) was limited to those with ≥16 years of education. It is also interesting to note that cancer death rates for women with ≥16 years of education are similar to those for women with 13–15 years of education. This pattern is largely driven by breast cancer death, for which the rates were higher among the most educated women than those with 13–15 years of education.

For diabetes and accidents, death rates increased significantly in the two less educated groups (≤12 and 13–15 years), except for diabetes in women with 13–15 years of education (Table 1 and Figure 1). For the most educated group (≥16 years), in contrast, rates significantly decreased for diabetes in women, while rates were stable for the remaining categories by sex and cause (diabetes and accidents) (Table 1 and Figure 1).

**Figure 2:**
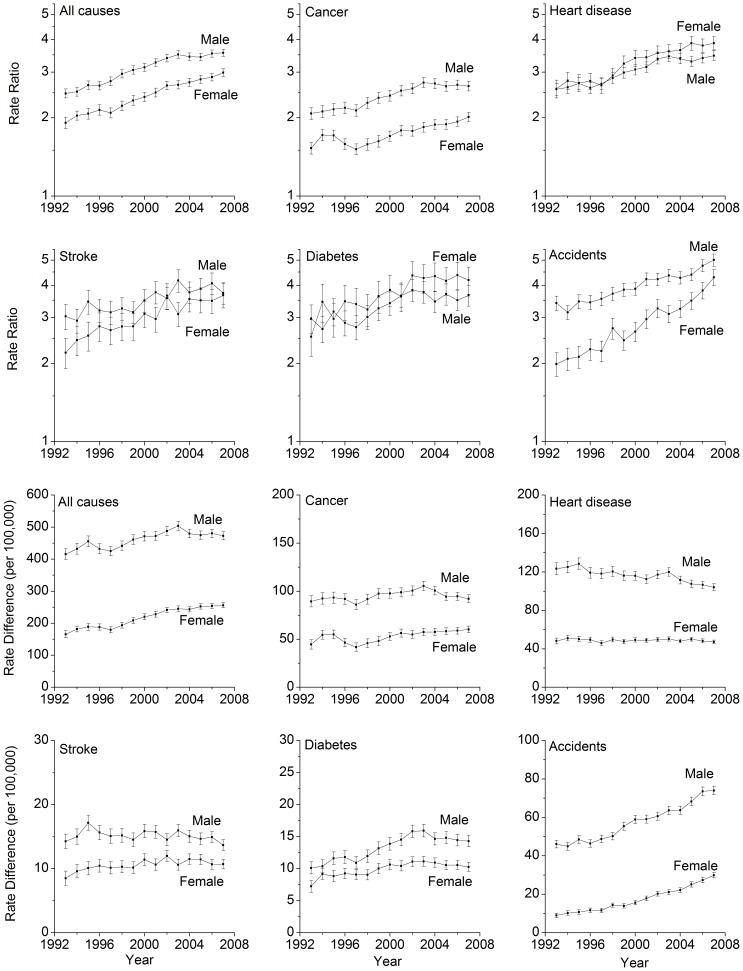
Temporal Changes of Educational Disparities in Mortality from All Causes and Five Major Causes in 26 U.S. States, 1993–2007.

Table 2 presents educational disparities in mortality from all causes combined and five major causes among men and women in 1993, 2001, and 2007. In men, the rate ratio of all-cause mortality comparing ≤12 years to ≥16 years of education increased from 2.5 (95% CI, 2.4–2.6) in 1993, 3.3 (95% CI, 3.2–3.4) in 2001, to 3.6 (95% CI, 3.5–3.7) in 2007. However, the increases of relative disparities appeared to have slightly slowed down since 2003 (Figure 2). With regard to absolute disparities, the rate difference (per 100,000 persons) in all-cause mortality between men with ≤12 and ≥16 years of education increased from 415.5 (95% CI, 399.1–431.9) in 1993 to 503.7 (95% CI, 489.4–517.9) in 2003 (data not shown), then slightly decreased to 472.7 (95% CI, 460.2–485.2) in 2007 (Table 2). In contrast to the observed patterns in men, both relative and absolute disparities in all-cause mortality among women continued to increase from 1993 through 2007. The rate ratio increased from 1.9 (95% CI, 1.8–2.0) to 3.0 (2.9–3.1) and the rate difference from 165.4 (95% CI, 154.5–176.2) to 256.2 (95% CI, 248.3–264.2) (Table 2 and Figure 2).

**Table 2:** Relative and Absolute Disparities in Mortality by Educational Attainment in 1993, 2001, and 2007.

	Male					Female				
	Rate (per 100,000)			Disparity		Rate (per 100,000)			Disparity	
	≤12 years	13–15 years	≥16 years	RR* (95% CI)	RD* (95% CI)	≤12 years	13–15 years	≥16 years	RR* (95% CI)	RD* (95% CI)
All causes										
1993	695.5	352.7	279.9	2.5(2.4,2.6)	415.5(399.1,431.9)	346.1	186.8	180.8	1.9(1.8,2.0)	165.4(154.5,176.2)
2001	680.0	306.5	207.9	3.3(3.2,3.4)	472.1(458.1,486.0)	379.8	186.7	151.7	2.5(2.4,2.6)	228.1(219.3,236.9)
2007	657.1	295.7	184.4	3.6(3.5,3.7)	472.7(460.2,485.2)	385.3	189.4	129.0	3.0(2.9,3.1)	256.2(248.3,264.2)
Cancer										
1993	172.2	96.0	82.9	2.1(2.0,2.2)	89.4(83.5,95.2)	129.1	84.5	91.7	1.4(1.3,1.5)	37.4(31.7,43.2)
2001	162.8	80.9	64.0	2.5(2.4,2.7)	98.8(94.0,103.5)	128.1	73.6	71.7	1.8(1.7,1.9)	56.4(52.3,60.6)
2007	148.0	72.7	55.9	2.6(2.5,2.8)	92.2(88.1,96.2)	119.4	69.1	59.2	2.0(1.9,2.1)	60.2(56.8,63.6)
Heart disease										
1993	201.7	102.7	78.2	2.6(2.4,2.7)	123.5(117.4,129.5)	78.6	35.1	30.3	2.6(2.4,2.8)	48.2(45.2,51.2)
2001	165.1	75.8	52.6	3.1(3.0,3.3)	112.5(108.1,116.9)	69.1	30.1	20.2	3.4(3.2,3.6)	48.9(46.7,51.0)
2007	146.2	68.0	42.1	3.5(3.3,3.6)	104.1(100.5,107.7)	63.7	27.7	16.4	3.9(3.7,4.1)	47.3(45.4,49.2)
Stroke										
1993	21.3	10.3	7.0	3.0(2.7,3.4)	14.3(13.1,15.4)	15.6	7.6	7.1	2.2(1.9,2.5)	8.5(7.4,9.6)
2001	21.4	8.4	5.7	3.8(3.4,4.2)	15.7(14.7,16.7)	16.0	7.2	5.4	3.0(2.7,3.3)	10.6(9.8,11.5)
2007	18.7	7.9	5.0	3.7(3.4,4.1)	13.7(12.8,14.5)	14.7	7.2	4.0	3.7(3.3,4.1)	10.7(9.9,11.5)
Diabetes										
1993	15.2	8.0	5.1	3.0(2.6,3.4)	10.1(9.2,11.1)	12.0	5.4	4.7	2.5(2.2,3.0)	7.2(6.3,8.2)
2001	20.0	9.6	5.5	3.6(3.3,4.0)	14.5(13.6,15.5)	14.4	6.2	4.0	3.6(3.2,4.1)	10.4(9.6,11.2)
2007	19.7	9.5	5.4	3.7(3.3,4.0)	14.3(13.4,15.2)	13.5	6.0	3.2	4.2(3.7,4.7)	10.3(9.6,11.0)
Accidents										
1993	65.2	27.7	19.1	3.4(3.2,3.6)	46.1(44.1,48.1)	18.1	10.0	9.1	2.0(1.8,2.2)	9.0(7.9,10.1)
2001	77.5	32.3	18.4	4.2(4.0,4.5)	59.1(57.0,61.2)	27.0	13.0	9.1	3.0(2.7,3.2)	17.9(16.7,19.1)
2007	92.5	39.0	18.5	5.0(4.7,5.3)	74.0(71.8,76.3)	38.9	17.9	9.1	4.3(4.0,4.6)	29.9(28.5,31.3)

Abbreviation: CI, Confidence Interval; RR, Relative Risk; RD, Rate Difference.

* ≤12 years of education Vs. ≥16 years of education.

For the five major causes listed in Table 2, increased relative disparities from 1993 to 2007 were observed among both men and women (with the confidence intervals overlapping for stroke and diabetes among men), with the largest increases for accidents, especially among women (RR = 2.0 [95% CI, 1.8–2.2] in 1993 and RR = 4.3 [95% CI, 4.0–4.6] in 2007). In contrast, absolute disparity patterns varied between genders and across the five major causes of death. Specifically, for cancer death, the rate difference between groups with ≤12 and ≥16 years of education remained stable among men but increased among women from 1993 to 2007; for heart disease, the rate difference decreased among men but remained stable among women; for stroke, the rate difference remained stable among men but increased among women; for diabetes and accidents, the rate differences increased among both men and women (Table 2). Notably, disparity patterns have slightly changed since 2003 for some specific causes. For example, both relative and absolute disparities have been slightly decreasing since 2003 for cancer among men; ratio ratios remained relatively stable since 2003 for stroke and diabetes among both men and women (Figure 2).

Table 3 shows the distributions of education and causes of death in 1993 and 2007. The proportion of highly educated persons (≥16 years of education) increased from 25.2% in 1993 to 29.9% in 2007 in men and from 20.8% to 31.3% in women. In contrast, the proportion of less educated persons (≤12 years of education) decreased from 51.4% to 45.2% in men and from 53.4% to 40.3% in women. Cancer and heart disease, the two leading causes of deaths among men and women aged 25–64 years, together comprised of 50% to 60% of the total deaths in each educational group (Table 3). From 1993 to 2007, the proportion of cancer deaths decreased among women in each educational group and among men with ≤12 years of education, but increased among men with 13–15 years and ≥16 years of education. During the same time period, the proportions of deaths from heart disease and stroke decreased among each gender and educational groups, except for stroke among men with 13–15 years and ≥16 years of education. In contrast, the proportions of deaths from diabetes and accidents increased among each gender and educational group, except for diabetes among women with ≥16 years of education (Table 3).

**Table 3:** Educational Distribution of Population and Cause of Death Fractions by Gender in 1993 and 2007.

	≤12 years			13–15 years			≥16 years		
	1993	2007	% change	1993	2007	% change	1993	2007	% change
Male									
Population fraction (%)	51.4	45.2	−12.1	23.4	24.9	6.4	25.2	29.9	18.7
Death fraction (%)									
Cancer	25.1	23.6	−6.0	24.5	25.7	4.9	28.5	31.5	10.5
Heart disease	27.6	22.8	−17.4	25.4	23.4	−7.9	25.4	23.4	−7.9
Stroke	3.0	2.9	−3.3	2.7	2.7	0.0	2.4	2.8	16.7
Diabetes	2.2	3.1	40.9	2.2	3.3	50.0	1.8	3.0	66.7
Accidents	9.4	13.0	38.3	9.7	12.2	25.8	7.5	9.0	20.0
Female									
Population fraction (%)	53.4	40.3	−24.5	25.7	28.4	10.5	20.8	31.3	50.5
Death fraction (%)									
Cancer	37.7	33.0	−12.5	43.3	37.4	−13.6	49.6	45.9	−7.5
Heart disease	21.4	17.1	−20.1	16.0	14.9	−6.9	13.0	12.8	−1.5
Stroke	4.5	3.9	−13.3	4.0	3.8	−5.0	3.7	3.1	−16.2
Diabetes	3.6	3.7	2.8	2.8	3.2	14.3	2.5	2.5	0.0
Accidents	4.8	8.3	72.9	6.5	8.8	35.4	6.2	7.0	12.9

**Figure 3:**
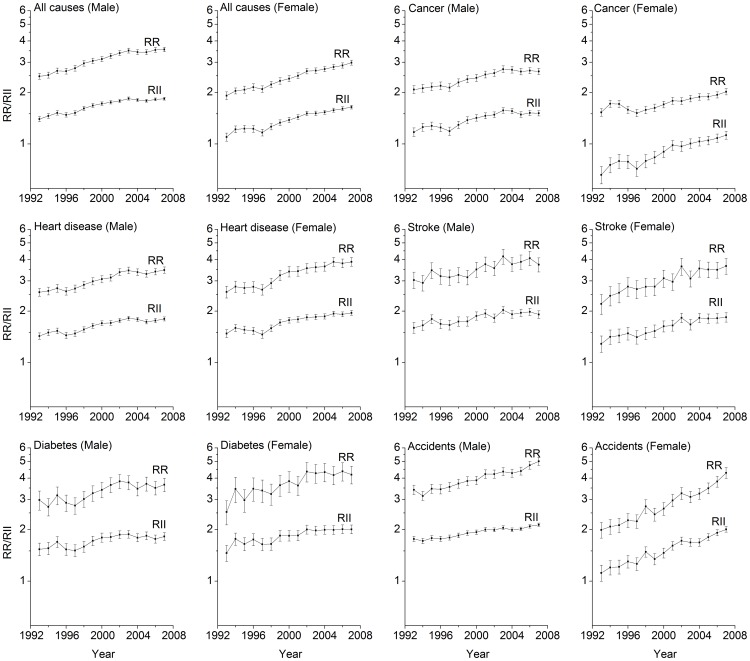
Temporal Trends in Relative Educational Disparities Measured by Relative Risk (RR) and Relative Index of Inequality (RII).

**Figure 4:**
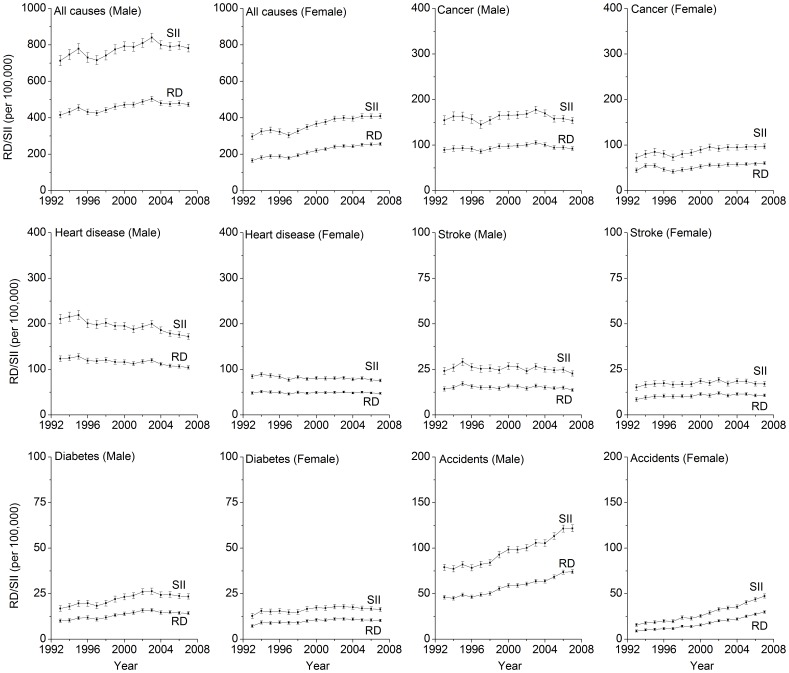
Temporal Trends in Absolute Educational Disparities Measured by Rate Difference (RD) and Slope Index of Inequality (SII).

Figure 3 and Figure 4 show the results from sensitivity analyses, which compared range measures (RR and RD) with summary measures (RII and SII) of disparity in measuring disparity trends. It shows that the resulting trends based on these two types of measures were almost identical for all the death causes considered in this study among both men and women.

Similar to what observed for all races combined, the differential trends that were more favorable to the most educated population were also observed in both non-Hispanic whites and blacks, although the cause-specific mortality trends for some educational groups were different between these two racial/ethnic groups (Tables S1 and S2 on the web). As a result, relative educational disparities in mortality from all causes and from the 5 major causes increased between 1993 and 2007 in both racial/ethnic groups, except for diabetes in non-Hispanic black men.

## Discussion

Our key finding is that relative disparities by education in all-cause mortality continued to increase from 1993 through 2007 among working-aged men and women in the 26 state reporting areas. Larger relative disparities from 1993 to 2007 also were observed for all five major causes of death considered in this study, with the largest increase for accidents, especially in women. Trends of absolute disparities varied largely by sex and cause of death. For example, the increases of absolute disparities in all-cause mortality continued from 1993 through 2007 in women, while the increases discontinued since 2003 in men.

From 1993 to 2007, the all-cause mortality rate decreased annually by 1.5% in men and by 0.7% in women, reflecting in part the progress in prevention and treatment of several major medical conditions, such as heart disease, cancer, and stroke [21], [22]. However, the mortality improvement was mainly concentrated in the most-educated populations. Those with the least education experienced either less progress or a worsening trend in mortality. For example, all-cause mortality in women with ≤12 years of education increased by 0.9% from 1993–2007. These differential trends that were more favorable to the most educated population resulted in increasing relative disparities between the most and least educated groups from 1993 through 2007. These findings add to previous reports that documented widening socioeconomic disparities in mortality since the 1960s [3]–[10]. Similar to our results, the middle-course assessment of U.S. Healthy People 2010 found that educational disparities remained unchanged or worsened for 106 of 109 outcome measures between 2000 and 2005 [23]. Widening socioeconomic disparities in mortality also have been reported in several Western and Northern European countries, including England and Norway [24]–[27].

The disparate educational mortality patterns for cancer, heart disease, and stroke may partly be explained by the historical differential changes (considering induction time from exposure to death) in some risk factors for these diseases. For example, the ratio of smoking prevalence between people with less than high school education and those with more than high school education increased from 1.3 in 1971–1974 to 2.3 in 1999–2002, due to smaller decreases in smoking among less educated people [28]. During the same time period, the prevalence of other major risk factors for heart disease and stroke, such as high blood pressure and cholesterol, remained much higher in less educated populations than in the most educated populations, despite improvements across all educational groups [28].

In addition to the differential changes in risk factors, worsening or persistent disparities in access to health care, health care quality, and early diagnosis could also have contributed to the widening of disparities in mortality. For example, from 1993 to 2006, the proportion of working-aged adults with no health insurance increased from 35% to 43% among those with less than high school education, while it remained at 7% among those with a college or above degree [29]. In addition, the 2010 National Healthcare Disparities Report (NHDR) found that over 85% (23 out of 26) of the measures of socioeconomic (indicated by income) disparities in access to health care and in health care quality remained unchanged or were worsening between 2001–2002 and 2007–2008 [30]. Between 2002 and 2008, the colorectal cancer screening rate among people aged ≥50 years remained about 20 percentage points higher in college graduates than in those with less than high school education [31].

Mortality rates from accidents increased rapidly from 1993 through 2007 in both men and women aged 25–64 years in the two lower educational groups, largely due to the dramatic increases in deaths from prescribed drug poisoning [32]. In addition, in concordance with the observed pattern for total accidents, death rates from drug poisoning have been increasing more quickly in women than in men [32]. This indicates drug poisoning may also be a major contributor to the increased educational disparities in total accidental deaths, although direct evidence still lacks. Similar to the U.S., increased poisoning deaths due to prescribed drugs were also found in some European countries, such as Finland [33]. Reasons for the substantial decrease in deaths from diabetes among females (restricted to the most educated group) are unclear. One speculation is that highly educated females may be more likely to seek and/or comply with diabetes treatment/management.

It is interesting to note that discrepant temporal patterns between relative and absolute disparities were observed for some causes, especially for heart disease in men. This highlights the relevance of using both relative and absolute measures in order to adequately assess health disparities. It is also noteworthy that for some diseases, trends of relative and absolute disparities have changed since 2003. Reasons for these patterns are unclear and further studies are needed.

Strengths of this study were twofold. First, the large number of deaths available in the NVSS mortality data enabled us to obtain precise estimates for each cause of death considered in this study. Second, the findings are not subject to sampling error because the NVSS mortality data are a complete count of all deaths registered in the U.S.

Despite the strengths mentioned above, our study results should be interpreted within a context of several limitations. First, using death rates as the only outcome measures, the extent to which mortality disparities are due to differences in incidence or in survival is not known. Second, range measures of disparity, i.e. rate ratio and rate difference between the lowest and highest education groups in this study, are unable to reflect the disparities caused by the change of educational compositions in the population. From 1993 to 2007, the proportion of highly educated persons increased in both men and women. However, results from sensitivity analyses indicate that educational composition change did not materially modify the trends of educational disparities in mortality from 1993 through 2007, as the observed disparity patterns indexed by range measures were almost identical to those indexed by summary measures (e.g. RII and SII).

The ability to generalize our findings to all age groups in the U.S. may be limited, due to the restricted analyses to ages of 25–64 years. Disparities in the elderly are expected to be narrower than those in younger populations, partly because of mortality selection (or survivor) effects and near-universal access to health care through Medicare among populations aged 65 years or above. Although the number of states included in our study (26 states) is also limited, we found that the 15-year-average (1993–2007) mortality rate and the distributions of education, sex and race/ethnicity in the population and among decedents in the 26 states are closely comparable to those in the entire U.S. population (data not shown, but available upon request). Thus, our findings may be applicable to the entire U.S., but are necessarily limited to those aged 25–64 years.

Imperfect measure of education is another potential contributor to the limitations. Educational information recorded on the death certificates was reported by next of kin. It has been found that proxy-reported education tends to be higher than self-reported, especially when differentiating between <12 and exactly 12 years of education [12], [34]. However, such misclassification would likely have little impact on our overall conclusions because we used a broad category for the least educated group including both <12 and exactly 12 years of education. Another source that may have affected our results is that different classification systems of education were used on the death certificate and in the CPS surveys. This difference in education classification between the numerator and denominator would have resulted in biased estimates of mortality rates, but would have no impact on our conclusions about the trends and widening disparities because both death certificates and CPS surveys kept their own classification methods constant during the entire study period. It is also noteworthy that educational attainment, the only indicator of socioeconomic position recorded on death certificates, does not fully capture the whole spectrum of socioeconomic disparities in mortality, as socioeconomic position is widely recognized as a multidimensional construct [35]. Another limitation related with socioeconomic status measurement is a lack of family level measures of education. The increase of educational assortative marriage (i.e. educational resemblance between spouses) in the U.S. may have led to increased disparities between educational groups [36].

Finally, the NCHS adopted ICD-10 in 1999 to classify and code the underlying cause of death. Because of changes in cause-of-death coding rules with the implementation of ICD-10, cause-of-death trends may be affected. Fortunately, none of the causes of death examined in this study were substantially affected. The change from ICD-9 to ICD-10 results in a slightly accelerated decline in death rates from heart disease and increase for accidents and diabetes (i.e., slightly fewer deaths were assigned to the declining heart disease trend and slightly more assigned to the increasing trends in accidents and diabetes beginning in 1999), and results in an attenuated decrease in rates for cancer and stroke (i.e., slightly more deaths assigned to declining trends in cancer and stroke beginning in 1999) [37]. This is unlikely to significantly affect our findings on disparities because the change is unlikely to have had a differential impact across educational groups. Also, it would have no impact on our findings regarding all-cause mortality, which is not affected by the change in disease classification.

In conclusion, mortality rates from all causes, cancer, heart disease, and stroke, continued to decrease among working-aged U.S. men and women from 1993 through 2007. However, relative mortality differences between the least and the most educated populations were increasing during this time period. This was due to comparatively slower progress or, in some cases, worsening trends among the least educated populations. Educational differentials in mortality continued to widen despite emphasis on reducing disparities in the U.S. Healthy People decennial initiatives.

## Supporting Information

Table S1 **Trends in Age-Standardized Death Rates from All Causes and Five Major Causes by Educational Attainment among Non-Hispanic Whites in 26 U.S. States, 1993–2007.**
(PDF)

Table S2 **Trends in Age-Standardized Death Rates from All Causes and Five Major Causes by Educational Attainment among Non-Hispanic Blacks in 26 U.S. States, 1993–2007.**
(PDF)

## References

[1] U.S. Department of Health and Human Services (2000). Healthy People 2010: Understanding and Improving Health. 2nd ed.

[2] U.S. Department of Health and Human Services, Office of Disease Prevention and Health PromotionU.S. Department of Health and Human Services, editor (2010). Healthy People 2020..

[3] Feldman JJ, Makuc DM, Kleinman JC, Cornoni-Huntley J (1989). National trends in educational differentials in mortality.. Am J Epidemiol.

[4] Schalick LM, Hadden WC, Pamuk E, Navarro V, Pappas G (2000). The widening gap in death rates among income groups in the United States from 1967 to 1986.. Int J Health Serv.

[5] Pappas G, Queen S, Hadden W, Fisher G (1993). The increasing disparity in mortality between socioeconomic groups in the United States, 1960 and 1986.. N Engl J Med.

[6] Steenland K, Henley J, Thun M (2002). All-cause and cause-specific death rates by educational status for two million people in two American Cancer Society cohorts, 1959–1996.. Am J Epidemiol.

[7] Steenland K, Hu S, Walker J (2004). All-cause and cause-specific mortality by socioeconomic status among employed persons in 27 US states, 1984–1997.. Am J Public Health.

[8] Meara ER, Richards S, Cutler DM (2008). The gap gets bigger: changes in mortality and life expectancy, by education, 1981–2000.. Health Aff (Millwood).

[9] Krieger N, Rehkopf DH, Chen JT, Waterman PD, Marcelli E (2008). The fall and rise of US inequities in premature mortality: 1960–2002.. PLoS Med.

[10] Hadden WC, Rockswold PD (2008). Increasing differential mortality by educational attainment in adults in the United States.. Int J Health Serv.

[11] Jemal A, Ward E, Anderson RN, Murray T, Thun MJ (2008). Widening of socioeconomic inequalities in U.S. death rates, 1993–2001.. PLoS One.

[12] Sorlie PD, Johnson NJ (1996). Validity of education information on the death certificate.. Epidemiology.

[13] Berkman LF, Macintyre S (1997). The measurement of social class in health studies: old measures and new formulations.. IARC Sci Publ.

[14] Albano JD, Ward E, Jemal A, Anderson R, Cokkinides VE (2007). Cancer mortality in the United States by education level and race.. J Natl Cancer Inst.

[15] World Health Organization (1977). International Statistical Classification of Disease and Related Health Problems: 9th Revision.

[16] World Health Organization (1992). International Statistical Classification of Disease and Related Health Problems: 10th Revision.

[17] Minino AM, Heron MP, Murphy SL, Kochanek KD (2007). Deaths: final data for 2004.. Natl Vital Stat Rep.

[18] Kominski R, Adams A, Census USBot, editor (1994). Educational Attainment in the United States: March 1993 and 1992..

[19] Kim HJ, Fay MP, Feuer EJ, Midthune DN (2000). Permutation tests for joinpoint regression with applications to cancer rates.. Stat Med.

[20] Rothman KJ, Greenland S, Lash TL (2008). Modern epidemiology.

[21] Jemal A, Ward E, Thun M (2010). Declining death rates reflect progress against cancer.. PLoS One.

[22] Roger VL, Go AS, Lloyd-Jones DM, Adams RJ, Berry JD (2011). Heart disease and stroke statistics–2011 update: a report from the American Heart Association.. Circulation.

[23] U.S. Department of Health and Human ServicesServices USDoHaH, editor (2007). Healthy people 2010: midcourse review..

[24] Mackenbach JP, Bos V, Andersen O, Cardano M, Costa G (2003). Widening socioeconomic inequalities in mortality in six Western European countries.. Int J Epidemiol.

[25] Strand BH, Groholt EK, Steingrimsdottir OA, Blakely T, Graff-Iversen S (2010). Educational inequalities in mortality over four decades in Norway: prospective study of middle aged men and women followed for cause specific mortality, 1960–2000.. BMJ.

[26] Shkolnikov VM, Andreev EM, Jdanov DA, Jasilionis D, Kravdal O (2012). Increasing absolute mortality disparities by education in Finland, Norway and Sweden, 1971–2000.. J Epidemiol Community Health.

[27] Mackenbach JP (2010). Has the English strategy to reduce health inequalities failed?. Soc Sci Med.

[28] Kanjilal S, Gregg EW, Cheng YJ, Zhang P, Nelson DE (2006). Socioeconomic status and trends in disparities in 4 major risk factors for cardiovascular disease among US adults, 1971–2002.. Arch Intern Med.

[29] Ahluwalia IB, Bolen J (2008). Lack of health insurance coverage among working-age adults, evidence from the Behavioral Risk Factor Surveillance System, 1993–2006.. J Community Health.

[30] Agency for Healthcare Research and QualityServices USDoHaH, editor (2011). 2010 National Healthcare Disparities Report..

[31] Rim SH, Joseph DA, Steele CB, Thompson TD, Seeff LC (2011). Colorectal cancer screening - United States, 2002, 2004, 2006, and 2008.. MMWR Surveill Summ.

[32] Centers for Disease Control and Prevention (2007). Unintentional poisoning deaths–United States, 1999–2004.. MMWR Morb Mortal Wkly Rep.

[33] Korhonen N, Niemi S, Parkkari J, Palvanen M, Kannus P (2011). Unintentional injury deaths among adult Finns in 1971–2008.. Injury.

[34] Rostron BL, Boies JL, Arias E, National Center for Health Statistics (U.S.) (2010). Education reporting and classification on death certificates in the United States.

[35] Braveman PA, Cubbin C, Egerter S, Chideya S, Marchi KS (2005). Socioeconomic status in health research: one size does not fit all.. JAMA.

[36] Schwartz CR, Mare RD (2005). Trends in educational assortative marriage from 1940 to 2003.. Demography.

[37] Anderson RN, Minino AM, Hoyert DL, Rosenberg HM (2001). Comparability of cause of death between ICD-9 and ICD-10: preliminary estimates.. Natl Vital Stat Rep.

