# Supplementary material for: Widening Educational Disparities in Premature Death Rates in Twenty Six States in the United States, 1993–2007
Source: PLoS One. 2012 Jul 20;7(7):e41560. doi: 10.1371/journal.pone.0041560 (PMC3401120; doi:10.1371/journal.pone.0041560)
Supplement: Table S1 — Trends in Age-Standardized Death Rates from All Causes and Five Major Causes by Educational Attainment among Non-Hispanic Whites in 26 U.S. States, 1993–2007. (PDF) [file pone.0041560.s001.pdf]

Table S1. Trends in Age-Standardized Death Rates from All Causes and Five Major Causes by Educational Attainment among Non-Hispanic Whites in 26 U.S. States, 1993-2007

	Non-Hispanic White Men			Non-Hispanic White Women		
	1993	2007	AAPC	1993	2007	AAPC
All Causes						
Education: All	495.3	420.2	-1.2*	268.6	248.1	-0.5*
≤12 years	620.6	649.8	0.1	311.2	386.4	1.5*
13-15 years	332.1	282.9	-1.0*	175.5	175.4	0.4*
16+ years	269.5	181.7	-2.8*	168.9	121.7	-2.1*
RR (≤12 vs 16+) (95% CI)	2.3 (2.2, 2.4)	3.6 (3.5, 3.7)		1.8 (1.7, 1.9)	3.2 (3.1, 3.3)	
RD (≤12 vs 16+) (95% CI)	351.2 (334.5, 367.8)	468.0 (454.0, 482.1)		142.4 (131.4, 153.3)	264.7 (255.7, 273.8)	
Cancer						
Education: All	136.6	101.7	-2.0*	115.9	89.4	-1.6*
≤12 years	163.0	148.8	-0.8	126.7	123.9	-0.2
13-15 years	94.6	71.3	-1.7*	84.0	66.3	-1.2*
16+ years	83.1	56.5	-2.9*	89.4	57.8	-2.9*
RR (≤12 vs 16+) (95% CI)	2.0 (1.9, 2.1)	2.6 (2.5, 2.8)		1.4 (1.3, 1.5)	2.1 (2.0, 2.2)	
RD (≤12 vs 16+) (95% CI)	79.8 (73.7, 86.0)	92.3 (87.7, 96.9)		37.3 (31.3, 43.3)	66.1 (62.2, 70.1)	
Heart Disease						
Education: All	143.7	94.1	-2.9*	50.5	36.2	-2.1*
≤12 years	181.3	143.9	-1.8*	61.1	59.9	-0.2
13-15 years	95.0	64.3	-2.6*	27.4	23.4	-0.8*
16+ years	72.7	40.4	-4.1*	22.1	14.0	-3.2*
RR (≤12 vs 16+) (95% CI)	2.5 (2.4, 2.6)	3.6 (3.4, 3.7)		2.8 (2.5, 3.0)	4.3 (4.0, 4.6)	
RD (≤12 vs 16+) (95% CI)	108.6 (102.5, 114.7)	103.4 (99.4, 107.5)		39.0 (36.3, 41.7)	46.0 (43.9, 48.0)	
Stroke						
Education: All	13.1	9.8	-2.5*	10.5	7.7	-2.2*
≤12 years	16.6	14.9	-1.1*	12.5	12.0	-0.2
13-15 years	9.1	6.7	-2.5*	6.2	5.7	-1.5*
16+ years	6.4	4.4	-3.5*	5.9	3.3	-3.9*
RR (≤12 vs 16+) (95% CI)	2.6 (2.3, 2.9)	3.4 (3.0, 3.8)		2.1 (1.8, 2.5)	3.7 (3.2, 4.2)	
RD (≤12 vs 16+) (95% CI)	10.2 (9.1, 11.3)	10.5 (9.6, 11.4)		6.6 (5.5, 7.7)	8.7 (7.9, 9.5)	
Diabetes						
Education: All	10.0	11.6	1.0*	7.9	6.9	-0.8*
≤12 years	12.6	17.9	2.5*	9.4	11.6	1.5*
13-15 years	7.2	8.5	1.9*	4.6	4.6	0.5
16+ years	4.7	4.7	-0.4	3.6	2.6	-2.3*
RR (≤12 vs 16+) (95% CI)	2.7 (2.3, 3.1)	3.8 (3.4, 4.2)		2.6 (2.1, 3.1)	4.5 (3.9, 5.2)	
RD (≤12 vs 16+) (95% CI)	7.9 (7.0, 8.9)	13.2 (12.3, 14.2)		5.8 (4.9, 6.6)	9.1 (8.3, 9.8)	
Accidents						
Education: All	43.0	62.8	2.9*	14.1	26.3	4.5*
≤12 years	60.6	103.7	4.1*	17.3	46.5	7.3*
13-15 years	26.2	40.3	3.5*	9.8	19.4	5.0*
16+ years	18.3	19.0	0.4	9.0	9.4	0.1
RR (≤12 vs 16+) (95% CI)	3.3 (3.1, 3.5)	5.4 (5.1, 5.8)		1.9 (1.7, 2.1)	4.9 (4.5, 5.3)	
RD (≤12 vs 16+) (95% CI)	42.3 (40.1, 44.4)	84.7 (81.7, 87.6)		8.2 (7.0, 9.4)	37.1 (35.1, 39.0)	

Abbreviations: AAPC, Average Annual Percent Change; RR, Rate Ratio; RD, Rate Difference; CI, Confidence Interval.

* $P<0.05$.
