# Supplementary material for: Widening Educational Disparities in Premature Death Rates in Twenty Six States in the United States, 1993–2007
Source: PLoS One. 2012 Jul 20;7(7):e41560. doi: 10.1371/journal.pone.0041560 (PMC3401120; doi:10.1371/journal.pone.0041560)
Supplement: Table S2 — Trends in Age-Standardized Death Rates from All Causes and Five Major Causes by Educational Attainment among Non-Hispanic Blacks in 26 U.S. States, 1993–2007. (PDF) [file pone.0041560.s002.pdf]

Table S2. Trends in Age-Standardized Death Rates from All Causes and Five Major Causes by Educational Attainment among Non-Hispanic Blacks in 26 U.S. States, 1993-2007

	Non-Hispanic Black Men			Non-Hispanic Black Women		
	1993	2007	AAPC	1993	2007	AAPC
All Causes						
Education: All	1090.2	793.2	-2.2*	526.1	439.7	-1.5*
≤12 years	1197.8	1017.3	-1.6*	596.5	554.9	-0.8*
13-15 years	588.5	462.2	-2.2	270.9	331.2	0.8
16+ years	539.9	330.9	-3.4	377.0	235.5	-2.3*
RR (≤12 vs 16+) (95% CI)	2.2 (1.9, 2.6)	3.1 (2.7, 3.5)		1.6 (1.3, 1.9)	2.4 (2.1, 2.6)	
RD (≤12 vs 16+) (95% CI)	657.9 (562.2, 753.6)	686.5 (626.5, 746.4)		219.5 (142.8, 296.2)	319.3 (286.4, 352.3)	
Cancer						
Education: All	240.3	170.4	-2.6*	154.5	126.4	-1.6*
≤12 years	260.0	216.5	-1.2*	164.1	145.4	-1.0*
13-15 years	137.0	101.7	-1.7*	93.2	105.9	0.3
16+ years	105.3	76.9	-2.3	146.0	86.1	-2.7*
RR (≤12 vs 16+) (95% CI)	2.5 (2.0, 3.1)	2.8 (2.4, 3.3)		1.1 (0.9, 1.4)	1.7 (1.5, 1.9)	
RD (≤12 vs 16+) (95% CI)	154.7 (126.1, 183.4)	139.6 (121.7, 157.5)		18.1 (0.0, 50.0)	59.3 (46.7, 71.8)	
Heart Disease						
Education: All	272.6	189.1	-2.5*	133.5	89.5	-2.8*
≤12 years	295.5	236.3	-2.0*	154.5	114.8	-2.1*
13-15 years	150.7	116.3	-1.2*	63.4	66.2	-0.1
16+ years	128.9	89.3	-3.9*	73.9	44.8	-3.0*
RR (≤12 vs 16+) (95% CI)	2.3 (1.9, 2.8)	2.6 (2.3, 3.1)		2.1 (1.6, 2.7)	2.6 (2.2, 2.9)	
RD (≤12 vs 16+) (95% CI)	166.6 (135.6, 197.6)	147.0 (129.0, 164.9)		80.6 (60.3, 100.8)	70.0 (61.2, 78.9)	
Stroke						
Education: All	46.5	35.1	-2.6*	32.5	24.3	-2.5*
≤12 years	51.6	45.6	-1.6*	36.7	30.8	-1.4*
13-15 years	22.4	17.6	-1.3	17.0	17.7	-0.3
16+ years	17.2	14.4	-2.9*	22.9	11.8	-2.6*
RR (≤12 vs 16+) (95% CI)	3.0 (2.1, 4.2)	3.2 (2.5, 4.0)		1.6 (1.1, 2.3)	2.6 (2.1, 3.3)	
RD (≤12 vs 16+) (95% CI)	34.4 (27.2, 41.6)	31.1 (26.2, 36.1)		13.8 (5.5, 22.1)	19.1 (15.5, 22.6)	
Diabetes						
Education: All	29.8	30.0	0.2	25.4	21.2	-1.9*
≤12 years	32.2	36.9	1.1	29.3	27.2	-1.3*
13-15 years	17.5	18.7	0.7	10.6	16.6	1.8
16+ years	14.8	16.9	-0.3	19.7	10.9	-2.4*
RR (≤12 vs 16+) (95% CI)	2.2 (1.5, 3.1)	2.2 (1.7, 2.7)		1.5 (1.0, 2.2)	2.5 (2.0, 3.2)	
RD (≤12 vs 16+) (95% CI)	17.4 (11.2, 23.5)	20.0 (15.2, 24.8)		9.6 (1.8, 17.4)	16.4 (13.0, 19.8)	
Accidents						
Education: All	77.1	71.8	-0.5	19.0	22.9	0.8*
≤12 years	90.9	93.0	0.1	23.1	30.9	1.7*
13-15 years	38.6	42.0	0.5	9.8	16.3	1.8*
16+ years	32.8	26.3	-1.4*	10.5	9.7	-2.0
RR (≤12 vs 16+) (95% CI)	2.8 (2.2, 3.5)	3.5 (3.0, 4.2)		2.2 (1.6, 3.1)	3.2 (2.5, 4.0)	
RD (≤12 vs 16+) (95% CI)	58.1 (48.9, 67.3)	66.8 (59.9, 73.7)		12.6 (8.5, 16.8)	21.2 (17.8, 24.6)	

Abbreviations: AAPC, Average Annual Percent Change; RR, Rate Ratio; RD, Rate Difference; CI, Confidence Interval.
* P<0.05.
